# Temporal Association of Acute Hepatitis A and *Plasmodium falciparum* Malaria in Children

**DOI:** 10.1371/journal.pone.0021013

**Published:** 2011-07-06

**Authors:** Peter Klein Klouwenberg, Philip Sasi, Mahfudh Bashraheil, Ken Awuondo, Marc Bonten, James Berkley, Kevin Marsh, Steffen Borrmann

**Affiliations:** 1 University Medical Center Utrecht, Utrecht, The Netherlands; 2 Kenya Medical Research Institute/Wellcome Trust Research Programme, Centre for Geographical Medicine Research – Coast, Kilifi, Kenya; 3 Muhimbili University, Dar es Salaam, Tanzania; 4 Department of Infectious Diseases, Heidelberg University School of Medicine, Heidelberg, Germany; Federal University of São Paulo, Brazil

## Abstract

**Background:**

In sub-Saharan Africa, *Plasmodium falciparum* and hepatitis A (HAV) infections are common, especially in children. Co-infections with these two pathogens may therefore occur, but it is unknown if temporal clustering exists.

**Materials and Methods:**

We studied the pattern of co-infection of *P. falciparum* malaria and acute HAV in Kenyan children under the age of 5 years in a cohort of children presenting with uncomplicated *P. falciparum* malaria. HAV status was determined during a 3-month follow-up period.

**Discussion:**

Among 222 cases of uncomplicated malaria, 10 patients were anti-HAV IgM positive. The incidence of HAV infections during *P. falciparum* malaria was 1.7 (95% CI 0.81–3.1) infections/person-year while the cumulative incidence of HAV over the 3-month follow-up period was 0.27 (95% CI 0.14–0.50) infections/person-year. Children with or without HAV co-infections had similar mean *P. falciparum* asexual parasite densities at presentation (31,000/µL vs. 34,000/µL, respectively), largely exceeding the pyrogenic threshold of 2,500 parasites/µL in this population and minimizing risk of over-diagnosis of malaria as an explanation.

**Conclusion:**

The observed temporal association between acute HAV and *P. falciparum* malaria suggests that co-infections of these two hepatotrophic human pathogens may result from changes in host susceptibility. Testing this hypothesis will require larger prospective studies.

## Introduction

In Africa, both *Plasmodium falciparum* malaria and hepatitis A virus (HAV) infections are common infections, especially in children [1], [2], although concurrent infections of these two highly prevalent human pathogens are likely to occur, knowledge about their prevalence and potential significance is poor. Since both pathogens target the hepatocyte as host cell for intracellular replication (mosquito-transmitted malaria parasites replicate silently in suitable hepatocytes before red blood cell invasion) direct or immunologically mediated interactions in concurrent infections could potentially escalate or inhibit progression of both infections.

Epidemiologically relevant interactions, though with contradictory findings, have previously been shown for *P. falciparum* malaria and hepatitis B (HBV). In a case-control study in The Gambia, the prevalence of HBV was significantly increased amongst children with severe *P. falciparum* malaria compared to matched controls [3]. Barcus et al found a prevalence of HBV infection of 24% in adult Vietnamese patients admitted with severe *P. falciparum* malaria, which was higher than the estimated prevalence of HBV in that area (10%) [4]. In contrast, a study in Papua New Guinea showed that female adults with the highest spleen rates of *Plasmodium* had the lowest prevalence of HBV infection, but there was no correlation with parasitemia [5]. Pasquetto et al showed a reduction of HBV replication rate, and ultimately, clearance of virions, in mice co-infected with *P. yoelii* [6]. It is also known that co-infections of other viruses such as Ebstein-Barr Virus [7], [8], and HIV [9] with *P. falciparum* affect the natural course of *P. falciparum* infections.

In this study we aimed to determine the temporal pattern of co-infection of *P. falciparum* malaria and acute HAV in a cohort of Kenyan children under the age of 5 years presenting with uncomplicated *P. falciparum* malaria. We focused on HAV since an initial viral screen (hepatitis A, B, C and D virus) of cases with elevated plasma concentrations of alanine aminotransferase (ALT; an established biomarker for the extent of liver cell damage) patients were positive for HAV, but not for any other hepatitis viruses.

## Results

A total of 222 children were included in this study. Forty patients (18.0%) had ALT plasma concentrations above the upper limit of normal for children (36 U/L) at one or more measurements at enrolment or during follow-up, and fifteen patients (6.7%) had ALT levels exceeding 100 U/L. All ten cases of HAV occurred in patient with ALT levels above 36 U/L (range 41–1295 U/L): eight patients had ALT levels ≥100 U/L, and two children had maximal ALT levels between 36–100 U/L (41 and 56 U/L). Demographic and laboratory data of HAV positive and negative children are shown in Table 1.

**Table 1:** Comparison of HAV positive and negative patients presenting with uncomplicated *P. falciparum* malaria.

	HAV positive (n = 10)	HAV negative (n = 68)	p-value
			
Age (months) (mean ± SD)	26.1±11.8	28.9±14.1	0.549#
Weight (kg) (mean ± SD)	10.5±2.13	10.8±2.41	0.734#
Sex (male/female) (number)	6/4	37/31	0.491§
Treatment (DHA-piperaquine/artemether-lumefantrine) (number)	8/2	48/20	0.534§
Baseline laboratory data			
Parasitemia (/µL) (geomean±95%CI)	37,000 (17,000–79,000)∞	48,000 (33,000–64,000)†	0.551*
Hemoglobin (g%) (mean ± SD)	8.1±1.8	9.2±1.4	0.031#
WBC count (/µL) (median, IQR)	8.7 (7.7–14.4)	9.0 (6.7–11.8)	0.774*
Creatinine (µmol/L) (mean ± SD)	38±6.5	43±9.6	0.144#
Total bilirubin (µmol/L) (median, IQR)	14.8 (12.3–23.6)	13.8 (8.8–24.6)	0.679*
ALT (U/L) at			
D0 (median (IQR))	92 (36–173)	29 (22–45)	0.016*
D3 (median (IQR))	64 (40–235)	28 (22–47)	0.028*
D7 (median (IQR))	81 (53–416)	21 (17–52)	0.012*
D28 (median (IQR))	27 (23–64)	23 (18–31)	0.385*
D42 (median (IQR))	21 (19–27)	24 (21–31)	0.270*
D84 (median (IQR))	26 (24–171)	27 (20–36)	0.599*
Recurrent *P .falciparum* infections (number)	5	19	0.374§

Geometric mean parasitemia at baseline were

∞ 31,000 (15,000–63,000) and

† 34,000 (24,000–47,000) when patients with high parasitemia are excluded.

# T-test,

*Mann-Whitney test,

§ chi square test.

We also tested the presence of IgM-HAV antibodies in 15 patients with normal ALT levels (≤36 U/L). These children were matched on treatment, study date, gender and age with the 15 patients having ALT levels ≥100 U/L. None of these patients showed a positive reaction for IgM-HAV. In addition, we tested an alternative hypothesis that *falciparum* malaria infections can lead to increased production of unspecific HAV-IgM [10] potentially causing false positive test results. We therefore measured 14 samples with the highest *P. falciparum* parasite density at baseline (of whom six children also belonged to the matched controls). The geometrical mean parasitemia in this subgroup was 196,000 parasites/µL (95% CI = 177,000–217,000; range = 164,000–350,000), and the median ALT level was 28 U/L (range 16–194 U/L). One patient with a parasitemia of 202,000/µL and a maximal ALT level of 41 U/L (case #10 in Table 2) showed a positive reaction. This indicated that elevated ALT plasma concentrations above 100 U/L had acceptable sensitivity (8/15, 53%) and high specificity (61/63, 97%), and levels above 36 U/L had low sensitivity (10/55, 18%) but excellent specificity (23/23, 100%) for detecting HAV infections in our cohort. As all available samples with ALT levels above the cut-off value of 36 U/L were measured, it is unlikely that we missed any additional HAV-positive cases.

**Table 2:** Summary of key laboratory parameters of patients who presented with uncomplicated *P. falciparum* malaria and subsequently found to have anti-HAV IgM indicating acute HAV infection.

Case #	Date of inclusion in study^1^	HAV detected (day)	Age (months)	ALT (U/L)						Baseline asexual parasite density (/µL)	Asexual *P. falciparum* parasite recurrence (day)
				Day 0	Day 3	Day 7	Day 28	Day 42	Day 84		
1	29/05/06	0	11	114	48	-	13	25	26	20,000	-
2	23/01/06	42	17	13	15	82	121	29	-	62,000	49
3	04/05/06	0	18	134	71	39	-	21	25	13,000	-
4	05/05/06	0	22	330	235	123	-	18	-	130,000	42
5	25/05/06	0	22	290	187	58	26	19	24	17,000	28
6	07/04/06	0	26	69	236	1295	45	21	-	126,000	-
7	18/05/06	0	28	118	327	80	27	-	42	19,000	35
8	06/12/05	84	38	25	44	-	-	21	559	20,000	-
9	11/10/05	0	28	40	56	-	-	17	-	14,000	-
10	05/05/06	0	52	41	29	-	27	29	23	202,000	56

1 One-third of patients were enrolled in May 2006.

Importantly, all but one case coincided with malaria (8 during first week and 1 on malaria reinfection; cases #1–7 and cases #9–10 in Table 2). The other case (case #8) had elevated ALT levels at day 84, indicating that a possible re-infection, if having occurred in this patient, could not have be detected due to end of follow-up. Thus, the number of HAV positive patients in this cohort of children in the 3-month follow-up period was 10 cases per 37.3 person-years (222 children with an average follow-up time of 2.0 months) resulting in a cumulative incidence of 0.27 (95% CI 0.14–0.50) infections per person-year, while during malaria there were 9 HAV-positive cases per 5.4 person-years, resulting in an incidence of 1.7 (95% CI 0.81–3.1) HAV infections per person-year. In the latter case, we calculated the incidence rate based on a 1-week time frame, that is, during and shortly after the malaria episode, and took into account all malaria episodes during baseline and all 73 recurrences that occurred during follow-up of the cohort of 222 children. The choice of a 1-week time frame thus resulted in an artificial saturation of the incidence rate (>1 infection per person-year) in a disease with 100% immunity. However, using this unit we obtained a realistic estimate for the incidence rate of HAV infections in children during the convalescence phase (0.27 per person-year), which is consistent with estimates that nearly all children in this part of Africa will have experienced an infection before the age of 5 years [2]. The proportion of children with elevated ALT levels differed significantly between baseline (60/222, 27.0%) and follow-up (9/89, 10.1%) (difference, 16.9%; 95%CI 7.6–26.3%; *P* = 0.001). A possible explanation could be the aggregation of measurements from baseline to day 7, because when only data from baseline were used, the difference ceased to be significant (39/222; difference, 7.5%, 95%CI −1.4%–16.3%; *P* = 0.119), possibly indicating that other factors such as treatment could have caused the additional increase in ALT levels.

Only one child with acute HAV infection showed clinical symptoms of the infection, i.e., jaundice; this patient had the highest ALT plasma concentration (case #6 in Table 2). None of the other children had clinical signs or symptoms suggestive of viral hepatitis infection, apart from fever, which is the main clinical symptom of malaria. Fever clearance times were similar between HAV positive and negative patients (1.0 versus 1.1 days, respectively).

Fifty percent (5/10) of patients with HAV infection had a *P. falciparum* malaria recurrence, compared to 35 percent (19/54) of HAV uninfected patients (p = 0.37). Of the five patients with a malaria recurrence in the HAV positive group, four were found to be HAV infected at baseline; the other patient had an HAV infection at day 42 and experienced a recurrent *P. falciparum* infection 1 week later. Four patients had a re-infection; the other sample could not be analyzed. Of the 19 HAV negative patients with a malaria recurrence, 17 had a re-infection, 1 had both recrudescent primary and secondary re-infections, and 1 sample could not be analyzed.

## Discussion

The present study describes a temporal association between *P. falciparum* malaria and acute HAV in a cohort of pediatric patients. We found that nine out of ten cases of acute HAV infections occurred simultaneously with *P. falciparum* malaria infections.

What are the alternative explanations that could have lead to a potential overestimation of the temporal association of active HAV infections with uncomplicated *P. falciparum* malaria? Firstly, overestimation of the association might have occurred if patients with HAV infections presented primarily for seeking relief for symptoms of HAV infection, and not of malaria. The baseline blood stage density in our study group was high (>37,000 asexual parasites/µL), far exceeding the pyrogenic threshold of 2,500 parasites/µL in this population and thus, minimizing over-diagnosis of malaria as an alternative explanation [11], [12]. Moreover, most HAV infections in children below the age of 5 years are known to be asymptomatic [13] and indeed, patients with co-infections did not differ in times to symptom clearance compared to patients with *P. falciparum* mono-infections. Secondly, *P. falciparum* malaria and/or hepatotoxic side effects of antimalarial drugs can also lead to elevated plasma concentrations of liver enzymes, indicating hepatocellular damage [14]. This could have led to an overestimation of the temporal association, as samples were selected based on ALT levels. However, patients with high and low baseline parasite densities had similar ALT levels and HAV infections clustered in patients with elevated ALT concentrations, but not in matched controls (matched on treatment, study date, gender and age). Thirdly, false positive results could have been caused by an induction of unspecific IgM antibody production leading to false positive anti-HAV IgM test results. We addressed this question by measuring IgM antibodies in 14 patients with the highest blood stage parasite density at baseline. This could possibly have led to misclassification bias. However, even if this is true and this patient is regarded to be HAV negative, we still find a significant incidence rate ratio. The risk of more false positive cases is limited, as the three highest parasitemias amongst the HAV positive children are 216,000/µL (the child who tested positive), 130,000/µL, and 125,000/µL. The latter two values are much lower than the parasitemia of the patient who tested positive and also lower than the parasitemia of the patient with the highest parasitemia in the HAV negative group (170,000/µL). In addition, the ALT levels of these patients were among the highest of the group (330 and 1300 UI/L) making an alternative diagnosis than acute HAV very unlikely. Fourthly, an overestimation of the association with malaria may have occurred, if anti-HAV IgM remains positive (long) after viral HAV particles have been cleared from the liver. However, the anti-HAV IgM response becomes undetectable usually within 6 months [15], but HAV RNA can be detected for more than 400–600 days after ALT peak [16], [17]. So even if anti-HAV IgM stays positive for a long time after acute infection, viral HAV particles will be residing in hepatocytes during that time period; therefore possibly increasing the likelihood to find concurrence of acute HAV and malaria. We could also rule out the possibility of an outbreak of HAV infection since patients were enrolled continuously from 2005 to 2006 and HAV cases did not cluster during a particular period of the year.

We found that HAV infections were a common cause for elevated plasma concentrations of an established biomarker of liver cell damage (ALT) in these patients. Children from the Kilifi district with *P. falciparum* malaria and elevated ALT concentrations (>100 U/L) had a 53% (8/15) chance of being co-infected with HAV, indicating that almost 50% of elevations could not be explained by HAV infections. This percentage is high and warrants consideration whenever determining the safety profile of antimalarial drugs. This study did not reveal any adverse clinical consequences of concurrent infections, except for a significant lower hemoglobin level. We have no ready explanation for this difference. Unlike *Plasmodium* infections HAV is not known to affect hemoglobin levels. One could speculate that *Plasmodium* infections in co-infected children were protracted and thus caused a slight additional drop in hemoglobin concentrations. Malaria patients co-infected with HAV were as likely as patients without HAV co-infections to clear clinical symptoms and parasites. By extension, this also indicates that most HAV infections were asymptomatic as expected [13]. There was only one HAV case with highly elevated ALT and clinical symptoms suggestive of acute hepatitis, which developed during the treatment of malaria.

The biological mechanism, if any, behind the observed temporal association remains elusive. For instance, *P. falciparum* liver stage and/or blood stage infections could, mediated by unknown immune mechanisms, promote HAV replication and thus, increase the chance to detect active HAV infections. Alternatively, coincidental HAV infection could facilitate parasite survival at the liver stage leading to increased numbers of infective merozoites and higher likelihood of subsequent pathogenic blood stage infection, in a similar way to the suggested mechanism for the observed association between malaria and HBV. In the study by Thursz, the increased incidence of severe malaria that was observed in chronic HBV carriers was explained by reduced expression of HLA class I molecules by hepatocytes during chronic HBV infection [3]. HLA class I molecules are important in the recognition and subsequent lysis of *P. falciparum* infected hepatocytes by cytotoxic T lymphocytes (CTLs). Thus, the lysing of parasite-infected hepatocytes by cytotoxic T lymphocytes (CTLs) may have been impaired in HBV carriers leading to an increased susceptibility to severe malaria. Whether similar mechanisms play a role in the association of acute HAV and falciparum malaria remains unclear.

In conclusion, we provide tentative evidence for a temporal association between *P. falciparum* and HAV infections in children living in an area of high *P. falciparum* and HAV transmission. Unfortunately, there is very limited information available about baseline HAV rates in Kenya, which is needed to draw stronger conclusions. We did not measure this; nevertheless we observed a temporal association between falciparum malaria and HAV. Prospective and larger studies are required to further elucidate the epidemiological interaction between these two important human pathogens. Our data also highlight the need for more comprehensive analyses of the complex interactions of prevalent infectious diseases in endemic countries that contribute to an unacceptably high disease burden.

## Materials and Methods

### Study site

The study was conducted at the Pingilikani study site of the Kenya Medical Research Institute/Centre for Geographic Medicine Research-Coast (CGMR-C). The Pingilikani study site is located 20 km south of Kilifi town in Kilifi district along the Kenyan coast [18]. *P. falciparum* transmission rates have been estimated to range between 22 and 53 infective bites per person per year [19]. Currently there is no data on the incidence of HAV or any other hepatitis viruses in this age group in Kilifi District.

### Study design and patients

This study was nested in an ongoing clinical trial to assess the efficacy and safety of dihydroartemisinin-piperaquine in comparison with artemether-lumefantrine in children with uncomplicated *P. falciparum* malaria [20] and Borrmann *et al* (unpublished data). Ethical approval for this study was granted by the Kenya National Ethics Review Committee, the Oxford Tropical Research Ethics Committee and the Ethics Committee of the Heidelberg University School of Medicine. Children aged 6–59 months presenting to the Pingilikani Health Centre with symptoms suggestive of uncomplicated malaria and a documented *P. falciparum* infection with an asexual parasite density of 2,000–200,000 parasites/µL, were recruited. Written informed consent was obtained from the parents. Patients were monitored daily until parasite and fever clearance was achieved. Active follow-up was on days 3 and 7, then weekly on days 14, 21, 28, 35, 42, 56, and 63 and 84. All subjects were seen on any day between day 0 and 84 upon request. ALT was measured routinely at baseline and at day 3, 7, 28, 42, 84, and at asexual parasite recurrence. Although ALT levels in patients with active HAV infection are typically 4–100 times elevated [21], we determined acute HAV infection status in all available samples with ALT levels of >36 U/L which is the upper limit of normal in children. In order to identify whether false positive tests were occurring at high parasite density, acute HAV infection status was also determined in children who had a baseline parasitemia of >150,000/µL. We used samples from baseline to measure anti-HAV IgM titers in any sample with elevated ALT from baseline up to day 7 as we assumed that the anti-HAV IgM levels would not significantly change during this period. For the other time-points (day 28, 42, and 84) we used blood samples from the respective study days.

### Laboratory procedures

Malaria infection was confirmed by Giemsa-stained thick smear [22]. We used a commercial ELISA kit to detect IgM antibodies to HAV (ELISA Kit for IgM Antibody to Hepatitis A Virus, BioChain, USA). The assay was performed according to the manufacturer's instructions.

### Statistical analysis

Analysis was carried out using Stata version 9.2 (Statacorp, Texas, USA). Data were entered and checked for inconsistencies by two different persons to reduce faulty entries. The Kolmogorov-Smirnov test was used to verify the normality of distribution of continuous variables. Comparisons between groups were performed using the t-test or two-tailed unpaired Mann-Whitney test for continuous variables evaluated as normally distributed and not-normally distributed, respectively. The chi-square test was used for comparisons between categorical variables. Parasitemia results were summarized using geometrical means and 95% confidence intervals. All the remaining continuous variables were reported with arithmetic means and standard deviation, or by the median and IQR. *P*<0.05 was considered statistically significant. Exact binomial confidence intervals were calculated to compare incidence rates.
